# Comparative Analysis of Elective Versus Emergency Cholecystectomy: A Study Using the Parkland Grading Scale, Nassar Scale, and G10 Score

**DOI:** 10.7759/cureus.78782

**Published:** 2025-02-09

**Authors:** Hugo Fernando Narvaez Gonzalez, Rebeca Perez Cabeza de Vaca, Emma Berenice López Pacheco, Omar López Osorio, Julian Vargas Flores, Yazmin Shantal Castillejos Marquez, Arcenio Luis Vargas Avila

**Affiliations:** 1 Gastrointestinal Endoscopy, Centro Medico Nacional 20 de Noviembre, Instituto de Seguridad y Servicios Sociales de los Trabajadores del Estado (ISSSTE), Mexico City, MEX; 2 School of Medicine and Health Sciences, Tecnologico de Monterrey, Monterrey, MEX; 3 Surgery, Hospital Regional General Ignacio Zaragoza, Instituto de Seguridad y Servicios Sociales de los Trabajadores del Estado (ISSSTE), Mexico City, MEX

**Keywords:** acute cholecystitis, biliary duct injury, elective general surgery, emergency surgery, intraoperative scales, laparoscopic cholecystectomy

## Abstract

Introduction

Laparoscopic cholecystectomy (LC) is a standard surgical procedure that general surgeons perform to treat acute cholecystitis. The presentation of this condition can vary in severity due to preoperative and intraoperative risk factors. Intraoperative scales such as the Parkland Grading Scale (PGS), Nassar Scale (NS), and G10 Score (G10S) evaluate these aspects. These scales help determine the severity of the condition, the need for conversion to open surgery, and the possibility of injury. However, their utility may differ between elective and emergency surgeries.

Methods

The objective of this study was to describe the clinical differences and intraoperative scales as well as the conversion rate to open surgery and the incidence of bile duct injury (BDI) in patients who underwent LC postoperatively at the Hospital Regional General Ignacio Zaragoza (HRGIZ) in Mexico City. An observational, descriptive, retrospective cross-sectional study was conducted. Patients over 18 years treated in the General Surgery Service of HRGIZ for LC between March 2022 and April 2023 were included. Pregnant patients and those undergoing other surgeries were excluded. The analyzed variables included age, gender, type of procedure, surgical time, scales (PGS, NS, G10S), conversion rate to open surgery, and BDI. Descriptive and inferential statistics were used for data analysis, including chi-square tests, Student's t-tests, and differences in proportions, utilizing VassarStats 2023 (Vassar College, Poughkeepsie, US) and Microsoft Excel v2401 (Microsoft Corp., Redmond, US).

Results

A total of 445 postoperative LC patients were identified. Based on the inclusion criteria, a sample of 364 patients was analyzed and classified into two groups: scheduled procedures (139, 38.2%) and emergency procedures (225, 61.8%). Both groups showed a female prevalence, with an average age of 55±16 years for scheduled procedures and 49±15 years for emergencies (p<0.05). No significant differences were found in surgical time, bleeding volume, or hospital stay between the groups. A higher number of LCs were performed as emergencies (205, 56.3%) compared to scheduled procedures (129, 35.4%) (p<0.0002). Of the total procedures, 4.3% required conversion to open surgery, and three patients (0.82%) presented with BDI. Regarding the evaluated scales, the PGS showed results of 2.51±1.42 in scheduled procedures compared to 3.49±1.34 in emergencies, the G10S score was 2.71±1.76 in scheduled procedures and 4.38±1.93 in emergencies, and the NS showed 1.91±0.92 in scheduled procedures and 3.02±0.90 in emergencies (p<0.05 for all). A higher percentage of BDI and a higher conversion rate to open surgery were noted in emergency procedures rather than scheduled procedures (3.02 vs. 1.37).

Conclusions

This study highlights the critical role of intraoperative scoring systems in predicting surgical difficulty and associated risks in LC. The significant differences in scores between elective and emergency surgeries reinforce their value in guiding surgical decision-making and tailoring approaches to individual patients. By integrating these scales into routine practice, surgeons can enhance outcomes, particularly in high-risk cases, while reducing complications such as BDI. Future research should focus on multi-institutional validation to further establish their utility in diverse clinical settings.

## Introduction

Laparoscopic cholecystectomy (LC) is a standard treatment for patients with symptomatic gallstones cholelithiasis [1]. These procedures have many complications, such as conversion, bleeding, and bile duct injury (BDI), which can vary depending on the complexity of the cholecystitis [2]. 

Although Strasberg’s critical view of safety (CVS), described in 1995, remains the preferred technique for safe dissection during cholecystectomy, some instances of inflammation can hinder the proper identification of anatomical structures, necessitating bail-out strategies to minimize complications [3-5].

For this reason, stratifying intraoperative difficulty through scoring systems incorporating preoperative and intraoperative factors is essential for devising strategies to optimize cholecystectomy outcomes [6].

Despite prior studies employing the Parkland Grading Scale (PGS), Nassar Scale (NS), and G10 Score (G10S), which focus on gallbladder characteristics, the presence of adhesions complicating dissection, and inflammation primarily in the lower third where the cystic duct and cystic artery are located, these scoring systems have typically been evaluated individually. To date, no study has comprehensively analyzed all three scales simultaneously to determine the risk of conversion to open surgery or BDI in elective and emergency procedures. Nonetheless, higher scores on these scales are associated with increased risks of conversion to open surgery and BDI [6-9].

This study analyzes the application of these three scoring systems in patients undergoing elective and emergency LC at a tertiary care hospital in Mexico City.

## Materials and methods

This observational, descriptive, and retrospective cross-sectional study, structured based on the Strengthening the Reporting of Observational Studies in Epidemiology (STROBE) checklist to enhance comprehension and adhere to best practices, was conducted in the General Surgery Service of the Hospital Regional General Ignacio Zaragoza (HRGIZ), Instituto de Seguridad y Servicios Sociales de los Trabajadores del Estado (ISSSTE) in Mexico City. The hospital database initially identified 445 patients who underwent LC between March 2022 and April 2023.

The inclusion criteria were adult patients (≥18 years) undergoing LC for symptomatic cholelithiasis or cholecystitis, with documented data on the PGS, NS, and G10S. The exclusion criteria included pregnant patients and those undergoing concurrent surgical procedures (e.g., umbilical hernia repair, liver biopsy) or requiring intraoperative cholangiography or bile duct exploration. After applying inclusion and exclusion criteria, 364 patients were included in the final analysis. The patients were stratified into two groups: elective surgery and emergency surgery.

The following variables were collected: demographic variables (age, gender), clinical variables such as surgical time, blood loss, length of hospital stay (LHS), intraoperative scores (PGS, NS, G10S), and outcomes such as bailout procedures, including fenestrated subtotal cholecystectomy (FSC), reconstituted subtotal cholecystectomy (RSC), anterograde cholecystectomy (ACH), conversion to open surgery, and postoperative complications (including BDI and conversion to open surgery).

Data were sourced from the hospital’s electronic medical records. This study's retrospective design was inherently susceptible to bias, including selection bias, due to the application of inclusion/exclusion criteria and information bias from reliance on existing medical records. The final analytical sample consisted of 364 patients.

Descriptive statistics were employed for continuous and categorical variables, such as means, standard deviations, and percentages. Inferential statistics included the chi-square test for categorical variables between elective and emergency groups and the Student’s t-test for continuous variables between groups. A p-value of less than 0.05 was considered statistically significant. Data analysis was conducted using VassarStats 2023 (Vassar College, Poughkeepsie, US) and Microsoft Excel v2401 (Microsoft Corp., Redmond, US), with double-checking of data entry and statistical calculations for quality assurance.

All data were anonymized, and no direct patient identifiers were used. Additionally, all authors declared no conflicts of interest. The surgical technique was aimed at completing cholecystectomy whenever possible, utilizing bailout procedures when necessary. Experienced surgeons and surgical residents performed all procedures. Based on the operationalization of the description and scoring of the PGS, NS, and G10S, we reviewed the scales from the original article with each of the resident physicians in training and the attending physicians who participated in the study to include them in the assessment of the patients.

## Results

A total of 445 postoperative LC patients were identified. Based on the inclusion criteria, a sample of 364 patients was analyzed and classified into two groups: elective procedures and emergency procedures. The data were rigorously collected and analyzed. In both elective and emergency surgeries, a higher proportion of female patients was observed with 139 (38.2%) and 225 (61.8%) cases, respectively, showing no statistically significant difference (p=0.7054). The mean age was 55±16 years for elective procedures and 49.32±15 years for emergencies (p<0.05).

The findings of this study are significant as no differences were found between the groups regarding surgical time, blood loss, or LHS. Emergency surgeries accounted for 205 cases (56.3%), while elective surgeries comprised 129 cases (35.4%) (p=0.0002). Clinical and sociodemographic characteristics are presented in Table 1. Regarding the type of procedures performed, the most common bail-out technique was laparoscopic reconstituted subtotal cholecystectomy. In contrast, other methods were performed less frequently, predominantly in emergency surgeries, without statistically significant differences between the groups (p=0.2802). A higher incidence of BDI and an increased conversion rate to open surgery were observed in emergency procedures compared to elective procedures (1.37% vs. 3.02%). Of all procedures, 4.3% required conversion to open surgery, and three patients (0.82%) experienced BDI, as described in Table 2. 

**Table 1:** Clinical and sociodemographic characteristics of the population * indicates statistical significance (p<0.5). SD: Standard deviation; LHS: Length of hospital stay; BDI: Biliary duct injury

Variables	Patients with cholecystectomy n=364		p-value
Type of surgery (%)	Elective surgeries n=139 (100)	Emergency surgeries n=225 (100)	<0.05*
Men (%)	44 (31.65)	67 (29.77)	0.7054
Women (%)	95 (68.34)	158 (70.22)	0.7054
Average age (SD)	55±16.21	49.317±14.73	0.00061*
Type of surgery (% of total patients n=364)	139 (38.18)	225 (61.81)	<0.0002*
Procedure time (mins ± SD)	114.85±50.71	112.69±43.55	0.67
LHS	4.36	4.39	0.95
BDI (%)	1 (0.004 )	2 (0.014)	>0.05

**Table 2:** Classification by type of procedure and its characteristics * indicates statistical significance (p<0.5); ^​​​a^ indicates chi-square test; ^†^ indicates conversion rate to open surgery in emergency cases (calculated from the total number of emergency cases); ^‡^ indicates overall patient conversion rate to open surgery (calculated from total patients, n=364). SD: Standard deviation

Elective and emergency surgeries (%)	Elective surgeries n=139 (100)	Emergency surgeries n=225 (100)	p-value
Type of surgery (% of total patients n=364)	139 (38.18)	225 (61.81)	<0.0002*
Type of procedure			
Laparoscopic cholecystectomy (%)	129 (35.4)	205 (56.3)	<0.0002*
Subtotal fenestrated cholecystectomy (%)	0 (0)	1 (0.27)	0.2802^a^
Reconstituted subtotal cholecystectomy (%)	4 (1.1)	8 (2.2)	
Fundus first cholecystectomy (%)	1 (0.27)	1 (0.27)	
Open cholecystectomy (%)	5 (1.3)	5 (1.3)	
Subtotal open cholecystectomy (%)	0 (0)	6 (1.7)	
Bleeding volume (cc ± SD)	117.55±266.97	123.67±230.61	>0.05
Surgery time (mins ± SD)	114.85±50.71	112.69±43.55	>0.05
Patients with conversion to open surgery ^†^	5	11	0.5592
Conversion rate (calculated based on totals by type of surgery) ^‡^	3.59	4.88	>0.05
Total patient conversion rate ^†^	1.37	3.02	>0.05

Evaluation of PGS, NS, and G10S

The PGS was applied in 97.2% of cases, while the G10S and NS were assessed in 73.3% and 37% of cases, respectively, according to the scores described in Tables 3-5. This analysis revealed a statistically significant disparity in the complexity of cholecystectomies performed as emergency procedures versus those electively. The data revealed a clear trend toward higher scores in the emergency group, suggesting a higher degree of surgical difficulty and potentially increased risk of complications.

**Table 3:** G10 Score GB: Gallbladder; BMI: Body mass index

Cholecystitis severity [9]	Score
Appearance	
Adhesion <50% of the GB	1
Adhesion >50% but GB partially buried	2
Completely buried GB	3
Distension/contraction	
Distended GB or contracted shrunken GB	1
Inability to grasp GB without decompression	1
Stone >1 cm impacted in Hartmann's pouch	1
Access	
BMI >30	1
Adhesions from previous surgery limiting surgery	1
Sepsis and complications	
Free bile or pus outside the GB	1
Fistula	1
Total possible	10

**Table 4:** Parkland Grading Scale GB: Gallbladder

Cholecystitis severity grade [10]	Description of severity
1	Normal appearing GB ("Robin's egg blue"). No adhesions present, completely normal GB.
2	Minor adhesions at the neck, otherwise normal GB. Adhesions restricted to the neck or lower body of the GB.
3	Presence of ANY of the following: hyperemia, pericholecystic fluid, adhesions to the body, distended GB.
4	Presence of ANY of the following: adhesions obscuring the majority of the GB 1-3 with abnormal liver anatomy, intrahepatic GB, or impacted stone (Mirizzi syndrome).
5	Presence of ANY of the following: perforation, necrosis, inability to visualize the GB due to adhesions.

**Table 5:** Nassar Scale

Nassar grades [11]	Gallbladder	Cystic pedicle	Adhesions
1	Lax, no adhesions	Clear and thin	Simple, at the neck and Hartmann's pouch
2	Mucocele, stone-filled	With fatty appendages	Simple, up to the gallbladder body
3	Deep gallbladder fossa, acute cholecystitis, fibrotic Hartmann's or obscured cystic duct pouch with adhesions to the bile duct or stone impaction	Normal anatomy, short, dilated, or obscured cystic duct	Dense, reaching the gallbladder fundus involving hepatic flexure or duodenum
4	Completely obscured, empyema/gangrene tumor	Impossible to identify	Dense, fibrous, involving the gallbladder, duodenum, or hepatic flexure, difficult to separate

Specifically, the PGS demonstrated a marked difference in the most frequent scores obtained: a score of 2 was most common in the elective group, reflecting relatively straightforward gallbladder anatomy and minimal inflammation; in contrast, the emergency group predominantly presented with a score of 5, indicating significantly more complex gallbladder pathology, such as severe inflammation, adhesions, or distortion. Similar trends were evident in the NS and G10S scores. The NS, which assesses gallbladder condition, cystic pedicle status, and adhesions, exhibited a most frequent score of 2 in elective cases, compared to a score of 3 in emergency cases, pointing to a greater degree of intraoperative adhesions and potentially challenging anatomy in emergencies. The G10S, which incorporates a broader range of intraoperative factors, including gallbladder appearance, accessibility, and presence of inflammation, showed a most frequent score of 2 among elective procedures and a significantly higher score of 6 among emergencies. This considerable difference underscores the heightened complexity and risk associated with emergency cholecystectomies. These findings corroborate the clinical observation that emergency procedures often involve more severe inflammatory states and significant surgical challenges than elective procedures.

The higher mean scores on the PGS (3.49 vs. 2.51), G10S (4.38 vs. 2.71), and NS (3.02 vs. 1.91) in emergency cases reinforce the utility of these scales in predicting complications, with a clearly defined significance (p<0.0001), as shown in Table 6.

**Table 6:** Intraoperative scoring system comparison in elective and emergency laparoscopic cholecystectomies ^a^ indicates chi-square test; ^b^ indicates Student's t-test. PGS: Parkland Grading Scale; G10S: G10 Score; NS: Nassar Scale; SD: Standard deviation

Elective and emergency surgeries	Elective surgeries n=139 (100%)	Emergency surgeries n=225 (100%)	p-value
PGS ± SD	2.51±1.42	3.49±1.34	<0.0001^b^
Degree 1	39	18	<0.0001^a^
Degree 2	47	41	
Degree 3	7	50	
Degree 4	20	36	
Degree 5	20	75	
G10S ± SD	2.71±1.76	4.38±1.93	<0.0001^b^
Score 1	24	13	
Score 2	27	24	
Score 3	22	30	
Score 4	4	24	
Score 5	7	29	
Score 6	5	32	
Score 7	1	14	
Score 8	1	9	
Score 9	0	0	
Score 10	1	0	
NS	1.91±0.92	3.02±0.90	0.0041^b^
Degree 1	14	4	0.006^a^
Degree 2	16	7	
Degree 3	9	14	
Degree 4	3	9	

## Discussion

Some evidence suggests that stratifying surgical difficulty using scoring systems can enhance strategic planning and surgical execution, ultimately leading to improved outcomes [6]. This has significant implications for patient care, allowing for better preoperative preparation and postoperative monitoring. For instance, a better understanding of the surgical difficulty can help in resource allocation and patient selection. Several preoperative and intraoperative severity scales have been evaluated to improve patient prognosis. However, while intraoperative scales do not identify suitable candidates for surgery, they help recognize cases requiring more advanced resolution techniques and closer postoperative monitoring [10].

The NS, a practical and convenient tool, evaluates three intraoperative characteristics: cystic pedicle and adhesions, using a score from 1 to 4. It is considered a significant factor, particularly for Nassar grades 4 and 5, associated with longer operative durations, higher conversion rates, and 30-day complications. Although grade 5 has been mentioned, less than 1% of patients fall into this category, and we did not include any patients in this study with Mirizzi syndrome of any grade or a fistula between the gallbladder and the skin or any intestinal segment [6,11].

In our study, despite 37% of patients being evaluated on an elective basis, we observed a higher proportion of patients with grade 3 (nine vs. 14 patients) and grade 4 (three vs. nine patients) in the emergency setting, with a statistically significant difference (p<0.006).

The PGS is reliable for determining gallbladder anatomy and various perioperative outcomes during inflammation. Its five categorical characteristics and interobserver correlation of 0.8210 demonstrate its reliability and accuracy for predicting outcomes following LC. This information can be crucial in deciding on the surgical approach and technique. The PGS has been validated by recognizing factors that increase the score, such as emergency cholecystitis, partial and open cholecystectomy, and bile leak rates [12].

Since this scale is applied at the outset of surgery, it is both faster and more practical, enabling the prediction of surgical difficulty through its evaluation system. However, compared to more sophisticated systems such as the Surgical Difficulty Grading System, it becomes evident that assessing the inflammatory state of Calot’s triangle, the presence of adhesions in this region, and the condition of scars within Calot’s triangle has a more significant impact on surgical difficulty due to the associated intraoperative and postoperative complications [13].

Published data regarding the use of the PGS exist in Mexico. A seven-month prospective analysis involving 105 patients demonstrated a correlation between the PGS results and surgical difficulty, particularly the need for conversion to open surgery and the performance of bailout procedures, such as subtotal cholecystectomy. The study found that grades 4 and 5 were statistically significant factors associated with conversion to open surgery and subtotal cholecystectomy [14].

From our perspective, the PGS is the most frequently analyzed scale, as there has been an effort to ensure that all patients undergoing LC are evaluated to predict complications. We found a statistically significant difference in patients with higher scores on the scale in the context of emergency surgery compared to elective surgery when performing the chi-square test (p<0.0001).

The G10S, a valuable and significant tool, focuses on four main intraoperative characteristics: gallbladder features (including the percentage of adhesions covering the gallbladder), whether it is distended or contracted, ease of access, the presence of peritoneal cavity fluid (whether purulent or biliary), and the existence of a cholecystoenteric fistula. With this scoring system, a score of <2 is considered easy, 2-4 moderate, 5-7 very difficult, and 8-10 extreme [8,9].

In a study conducted in South Florida analyzing 117 patients for LC, the G10S scores were classified using a binary logistics model and risk factors, resulting in an overall mean score of 2.3±1.5. This score significantly increased as severity progressed, with a mean value of 5.5±0.51 for complex cases (p=0.0001) and an area under the curve (AUC) of 0.79, with a 3.71±1.4 for open conversions (p=0.0001). All patients undergoing LC were assigned a G10 value, where gallbladder surgery was considered easy if the score was <2, moderate (2 to ≤ 4), complex (5 to ≤7), and extreme (8 to ≤10). All 10 risk factors were analyzed using a binary logistics model, identifying significant risk factors. The multivariate analysis indicated the presence of free bile or pus outside the gallbladder and fistula as substantial risk factors, with an odds ratio (OR) of 5.1 and 15.8, respectively [15].

In contrast to our study, operative duration did not show statistically significant differences, as some surgeries classified as elective involved patients who had been on a waiting list due to the high demand for operating rooms. Classifying them as elective ensured guaranteed surgical time, which explains why surgical duration and intraoperative blood loss did not show significant differences between the two groups. Furthermore, some patients required admission from the emergency department for continued medical management until their elective surgery, which extended their LHS. A systematic review of retrospective, prospective, and systematic review studies was performed in Australia, with 30 studies including 108,472 patients. They found that older age, male gender, previous abdominal surgeries, acute cholecystitis, symptom duration of more than 72 hours, prior history of acute cholecystitis, C-reactive protein value, Conversion from Laparoscopic to Open Cholecystectomy (CLOC) score of more than 6, G10S score of more than 3, diabetes, obesity, emergency LC, and urgent admissions are significant risk factors for conversion of LC to open cholecystectomy [16]. The CLOC score, which incorporates preoperative variables for cholecystectomy, allows the identification of patients at a six-fold increased risk of conversion to open cholecystectomy [17]. Besides, it is mentioned that the score's strong correlation with operative difficulty and BDI has important clinical implications. It allows for risk-based patient selection: low-risk patients (CLOC ≤6) can be treated in ambulatory settings and used for training, whereas high-risk patients (CLOC >6) require experienced surgeons and inpatient care to mitigate complications [18]. Given that the CLOC scale parameters are exclusively preoperative, we elected not to include them in this analysis. However, incorporating this scale in subsequent studies, alongside intraoperative assessment, would facilitate a more comprehensive evaluation for managing acute cholecystitis in surgical trainees. Such an approach would improve the efficacy of decision-making and resource allocation for acute cholecystitis cases within our teaching hospital.

Strengths and limitations of the study

This is the first study conducted in Mexico involving many patients, evaluating three of the most commonly used intraoperative scales in elective and emergency laparoscopic cholecystectomy settings. However, it is a single-center, retrospective study, and not all patients were assessed using these scales. Despite these limitations, the use of these scales has become more frequent, aiming to develop improved treatment strategies, particularly for patients at higher risk of conversion who may require alternative procedures, and to reduce the risk of BDI.

## Conclusions

This study comprehensively evaluates three commonly used intraoperative scoring systems in elective and emergency laparoscopic cholecystectomy settings. Our findings highlight the utility of these scales in stratifying surgical difficulty, identifying higher scores as significant predictors of conversion to open surgery and BDI, particularly in emergency procedures. This analysis underscores the value of standardized intraoperative grading systems in improving outcomes in laparoscopic cholecystectomy. The higher difficulty scores observed in emergency surgeries highlight the need for robust preoperative planning and implementing bail-out strategies in challenging cases. The PGS, NS, and G10S proved instrumental in stratifying surgical risk, demonstrating their potential to inform clinical practice. Expanding their use in multicenter and prospective studies will allow a more comprehensive understanding of their role in enhancing patient care and surgical efficiency.
